# Rationale and design of the British Heart Foundation (BHF) Coronary Microvascular Function and CT Coronary Angiogram (CorCTCA) study

**DOI:** 10.1016/j.ahj.2019.11.015

**Published:** 2020-03

**Authors:** Novalia P Sidik, Margaret McEntegart, Giles Roditi, Thomas J Ford, Michael McDermott, Andrew Morrow, John Byrne, Jacqueline Adams, Allister Hargreaves, Keith G Oldroyd, David Stobo, Olivia Wu, Claudia-Martina Messow, Alex McConnachie, Colin Berry

**Affiliations:** a West of Scotland Heart and Lung Centre, Golden Jubilee National Hospital, Glasgow, UK; b British Heart Foundation Glasgow Cardiovascular Research Centre, Institute of Cardiovascular and Medical Sciences, University of Glasgow, Glasgow, UK; c Glasgow Royal Infirmary, Glasgow, UK; d University of New South Wales, Sydney, Australia; e Forth Valley Royal Hospital, Larbert, UK; f Health Economics and Health Technology Assessment, Institute of Health and Wellbeing, University of Glasgow, Glasgow, UK; g Robertson Centre for Biostatistics, Institute of Health and Wellbeing, University of Glasgow, Glasgow, UK

## Abstract

Microvascular and/or vasospastic anginas are relevant causes of ischemia with no obstructive coronary artery disease (INOCA) in patients after computed tomography coronary angiography (CTCA).

**Objectives:**

Our research has 2 objectives. The first is to undertake a diagnostic study, and the second is to undertake a nested, clinical trial of stratified medicine.

**Design:**

A prospective, multicenter, randomized, blinded, sham-controlled trial of stratified medicine (NCT03477890) will be performed. All-comers referred for clinically indicated CTCA for investigation of suspected coronary artery disease (CAD) will be screened in 3 regional centers. Following informed consent, eligible patients with angina symptoms are enrolled before CTCA and remain eligible if CTCA excludes obstructive CAD.

Diagnostic study: Invasive coronary angiography involving an interventional diagnostic procedure (IDP) to assess for disease endotypes: (1) angina due to obstructive CAD (fractional flow reserve ≤0.80); (2) microvascular angina (coronary flow reserve <2.0 and/or index of microvascular resistance >25); (3) microvascular angina due to small vessel spasm (acetylcholine); (4) vasospastic angina due to epicardial coronary spasm (acetylcholine); and (5) noncoronary etiology (normal coronary function). The IDP involves direct invasive measurements using a diagnostic coronary guidewire followed by provocation testing with intracoronary acetylcholine. The primary outcome of the diagnostic study is the reclassification of the initial CTCA diagnosis based on the IDP.

Stratified medicine trial: Participants are immediately randomized 1:1 in the catheter laboratory to therapy stratified by endotype (intervention group) or not (control group). The primary outcome of the trial is the mean within-subject change in Seattle Angina Questionnaire score at 6 months.

Secondary outcomes include safety, feasibility, diagnostic utility (impact on diagnosis and certainty), and clinical utility (impact on treatment and investigations). Health status assessments include quality of life, illness perception, anxiety-depression score, treatment satisfaction, and physical activity. Participants who are not randomized will enter a follow-up registry. Health and economic outcomes in the longer term will be assessed using electronic patient record linkage.

**Value:**

CorCTCA will prospectively characterize the prevalence of disease endotypes in INOCA and determine the clinical value of stratified medicine in this population.

## Graphical abstract

**Figure:**
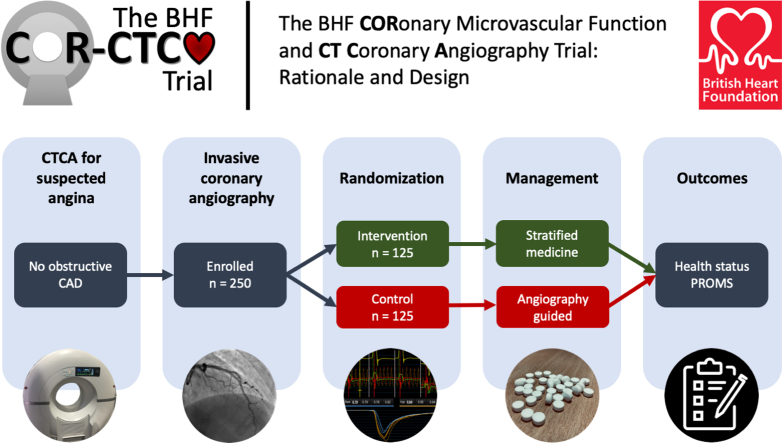

## Background

### Epidemiology

Ischemic heart disease (IHD) is a leading global cause of premature morbidity and death.1., 2. In many countries, the increase in longevity and improvements in IHD mortality have plateaued.1., 2. Recent consensus IHD guidelines reflect the diverse spectrum and etiopathogenesis of patients with chronic coronary syndromes.^3^ These include a continuum of coronary atherosclerosis and disorders of coronary vasomotion, including microvascular angina and vasospastic angina. Ischemia with no obstructive coronary artery disease (INOCA) is increasingly recognized and may be caused by transient and/or sustained impairments in supply-demand of myocardial perfusion.4., 5., 6. Coronary vascular dysfunction may be structural and/or functional and involve the coronary artery and/or its microcirculation.6., 7. Epicardial coronary heart disease (CHD) occurs more often in men,^8^ whereas functional disorders (microvascular angina and vasospastic angina) are more common in women.^9^

### Diagnosis using anatomical imaging of coronary artery disease

Following recent randomized trials,8., 10., 11., 12., 13. diagnostic imaging using computed tomography coronary angiography (CTCA) is recommended as a first-line test for the assessment of stable chest pain in patients with no prior history of coronary artery disease (CAD).14., 15., 16., 17. The Scottish Computed Tomography of the Heart (SCOT-HEART) trial reported that, among patients referred to a cardiology chest pain clinic with suspected stable angina, CTCA added to standard care clarified the diagnosis of CHD and altered subsequent management.^8^ At 5 years, CTCA-guided management added to standard care reduced the rate of death from CHD or nonfatal myocardial infarction (MI).^18^ On the other hand, compared with standard care, anginal symptoms and quality life at 6 weeks and 6 months were worse in the CTCA-guided group.^19^ Several factors may be relevant. One explanation could be that, in the CTCA group, in patients who had microvascular angina and/or vasospastic angina, exclusion of angina due to CHD resulted in discontinuation of angina therapy by protocol which in turn led to a deterioration in anginal symptoms and quality of life. This theme is reflected by our observations in clinical practice relating to patients with persistent, unexplained anginal symptoms following CTCA-guided management. Given that only 1 in 5 patients with recent onset angina has obstructive CAD, this knowledge gap is relevant. None of the landmark CTCA trials involved systematic evaluation of non–flow-limiting CAD and coronary vasomotion8., 10., 11., 12., 13., 19.; hence, the prevalence of coronary vascular dysfunction in the majority of patients with angina (or ischemic symptoms) and no obstructive CAD (INOCA) is unknown.

### Stratified medicine: evidence of endotypes linked to treatment strategies

The Coronary Microvascular Angina (CorMicA) trial^9^ provided new insights into the prevalence of microvascular angina and vasospastic angina in patients selected for invasive coronary angiography. The CorMicA investigators prospectively enrolled 391 patients referred for clinically indicated coronary angiography in a regional center during a 12-month period. Almost half of this population (n = 185; 47%) had no obstructive CAD. One hundred and fifty-one subjects entered the randomized trial, and those who had obstructive CAD (n = 206; 53%) and were therefore ineligible for randomization entered a registry. CorMicA involved a 1:1 randomized, blinded, sham-controlled, parallel-group, clinical trial of stratified medicine versus standard angiography-guided management. Stratified medicine involved adjunctive tests of coronary vascular function to identify disease endotypes with linked medical therapy. Compared to standard care, the stratified intervention changed the initial diagnosis based on coronary angiography in half of the participants in the intervention group and was associated with directionally consistent improvements in angina, quality of life, and treatment satisfaction at 6 months. CorMicA was positioned downstream in the care pathway in patients selected for invasive management. Whether or not endotypes, such as microvascular angina and/or vasospastic angina, might be common and clinically relevant in a population of patients presenting with stable angina in the Chest Pain Clinic setting is unknown. The Chest Pain Clinic represents the point-of-care linking referrals from primary care with cardiology in secondary care.

### Rationale

Several studies have addressed the prevalence of microvascular angina and vasospastic angina.9., 20., 21. However, the participants in these studies had been referred and selected for invasive coronary angiography which takes place downstream in the care pathway. The prevalence data from these studies may not accurately reflect the true prevalence of vasomotion disorders. In a comparatively unselected population of all-comers with chest symptoms and a lower burden of cardiovascular risk factors, vasomotion disorders may, potentially, be much more common than obstructive CAD. In the United Kingdom, anatomical imaging using CTCA is recommended as the first-line test for the evaluation of recent-onset chest pain suspected to be due to CHD.15., 16. The Chest Pain Clinic setting provides an ideal opportunity to assess for the prevalence of these conditions in a relatively unselected population, upstream in the care pathway.

Stratified medicine is the identification of key subgroups of patients (endotypes) within a heterogeneous population, these endotypes being distinguishable by distinct mechanisms of disease and/or responses to therapy.^22^ The CorMicA trial highlighted the potential for stratified medicine to benefit patients with angina. The strategy is now supported by a Class IIA practice guideline recommendation from the European Society of Cardiology.^17^ In CorCTCA, we now propose a randomized controlled trial to assess whether stratified medicine is informative and clinically useful in patients with angina and no obstructive CAD as determined by CTCA.

## Study design and methods

### Aim

Our first aim is to assess the prevalence of disease endotypes in patients with angina and no obstructive CAD classified by CTCA. Disease endotypes will be prospectively assessed using an interventional diagnostic procedure (IDP) including tests of coronary vascular function during invasive angiography. Our second aim is to assess the effect of a novel clinical strategy, stratified medicine guided by the IDP, on diagnosis, treatment, and well-being. The participants will be randomized before the IDP to prevent any bias by knowing the treatment group allocation. The participants will be randomly allocated into 2 groups: the intervention group (IDP disclosed, stratified medicine) or the control group (IDP not disclosed/sham, standard angiography-guided management).

### Hypothesis

In patients with angina in whom obstructive CAD has been excluded by CTCA, microvascular angina and vasospastic angina are prevalent. A systematic assessment of coronary vascular function using invasive coronary angiography and adjunctive tests of coronary vascular function will reclassify the diagnosis leading to changes in treatment and improvements in well-being as compared to decisions based on CTCA alone. We hypothesize that clarification of the diagnosis to rule in or rule out disease endotypes, increasing the certainty of the diagnosis, will help clinicians make informed therapy decisions. Finally, we hypothesize that stratified medicine will improve patient well-being and health care resource utilization.

### Objectives

Our research includes 2 primary objectives. The first is to undertake a diagnostic study, and the second is to undertake a nested, randomized, controlled, trial in participants enrolled into the diagnostic study.

#### Primary objective of the diagnostic study

The primary objective is to prospectively determine the prevalence of coronary endotypes in an INOCA population as classified by a CTCA. The primary outcome of the diagnostic study reflects the reclassification of the initial diagnosis using diagnostic tests of coronary vascular function. The primary outcome is determined by the reclassification of the following diagnostic groups (endotypes):

1. Angina due to obstructive CAD (fractional flow reserve [FFR] ≤0.80);
2. Microvascular angina (coronary flow reserve [CFR] <2.0 and/or index of microvascular resistance [IMR] >25);
3. Microvascular angina due to spasm (acetylcholine testing);
4. Vasospastic angina due to coronary spasm (acetylcholine testing);
5. Noncoronary etiology (normal coronary function).

The reclassification decision (yes/no) will be based on the results of the IDP versus the diagnosis based on CTCA. The primary analysis is the between-group comparison of the reclassification rate using logistic regression, adjusted for baseline factors associated with the likelihood of reclassification of the initial diagnosis.

#### Primary objective of the trial

The primary objective of the clinical trial is to determine whether stratified medicine, including disclosure of the coronary function findings with linked changes in management, leads to patient benefits. The primary outcome is the within-subject change at 6 months from baseline for the domains of the Seattle Angina Questionnaire (SAQ).

#### Secondary objectives

The secondary objectives will gather information on health status, physical activity, and health and economic outcomes.

1. Compare health status using the SAQ, the EuroQol 5-domain health-related quality of life questionnaire, the Illness Perception Questionnaire, the Patient Health Questionnaire-4 for anxiety/depression, and the Treatment Satisfaction Questionnaire for Medication between the intervention and control groups at baseline and during follow-up (6, 12, and 24 months or close-out);
2. Compare functional status (Duke Activity Status Index) and physical activity levels (International Physical Activity Questionnaire–Short Form) between the intervention and control groups at baseline and during follow-up;
3. Compare longer-term health outcomes and resource utilization including episodes of care and prescriptions between the intervention and control groups using electronic record linkage;

#### Tertiary (scientific) outcomes

1. Measure circulating biomarkers of cardiovascular function and inflammation;
2. Investigate pathophysiology including the relationships between (1) coronary calcium score, (2) shear stress, and parameters of coronary vascular function;
3. Develop novel biomarkers (imaging, blood, etc) of coronary vascular function.

### Study design

CorCTCA includes a prospective, diagnostic study and nested, 1:1 randomized, sham-controlled, parallel-group, blinded clinical trial.

### Setting

**Figure 1:**
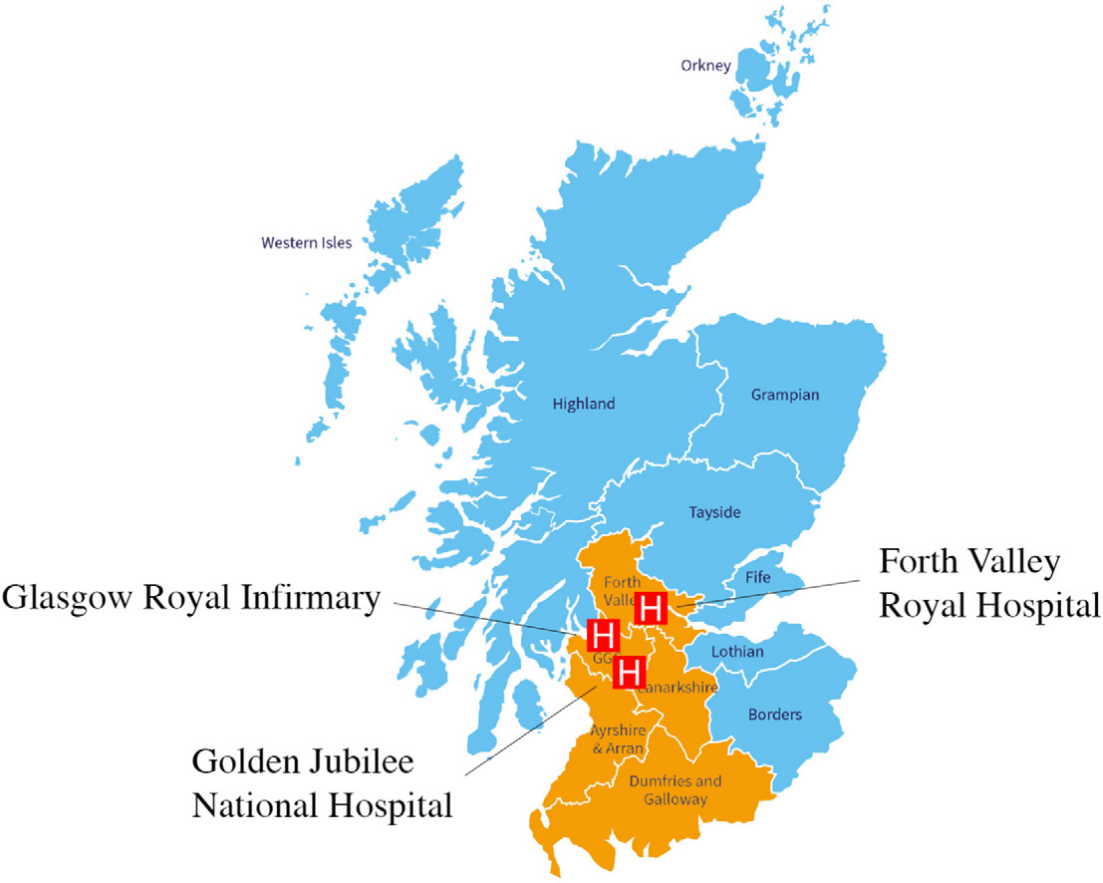
Recruitment sites for CorCTCA. Three recruiting hospitals with a catchment area which covers West and Central Scotland (labeled in orange).

Participants will be screened and enrolled at the point of care for CTCA at 3 or more hospitals in West and Central Scotland (population ~2.5 million) (Figure 1). Screening is initially performed by review of the electronic radiology referrals for CTCA. The subjects should have anginal symptoms and be referred by their attending cardiologist for clinically indicated CTCA in line with the contemporary practice guidelines.14., 15., 16., 17.

The sites include a regional cardiothoracic center, a large urban hospital, and a district general hospital. The geographies include socially diverse populations from urban and rural communities.

#### Eligibility criteria

**Figure 2:**
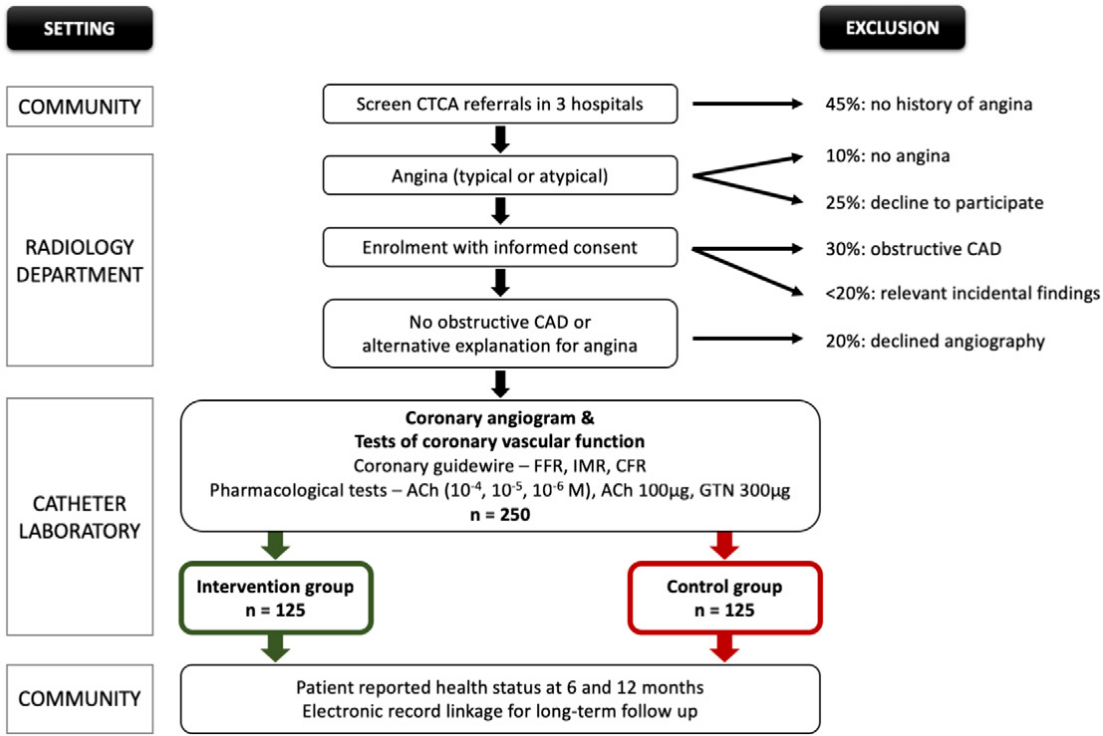
Schematic study design: flow diagram. *ACh*, acetylcholine.

To mitigate the possibility of bias through knowledge of the CTCA findings, the decision to enroll patients will be made before the CTCA (Figure 2). Patients referred for CTCA will be invited to give informed consent and complete the Rose Angina^23^ and Seattle Angina Questionnaires.^24^ The participants' responses disclosed in these questionnaires will then be assessed against the eligibility criteria to confirm a history of anginal symptoms. Participants who report symptoms of angina and fulfill the eligibility criteria will then be invited to complete the other health questionnaires before CTCA. By completing the questionnaires before CTCA, the participants will be unaware of the imaging results which therefore cannot influence the patients' responses.

The inclusion criteria are:

1. Age ≥ 18 years.
2. Symptoms of angina or angina-equivalent informed by the Rose Angina questionnaire.
3. Intermediate or no obstructive coronary disease, that is, no coronary stenosis >70% in an artery >2.5 mm, as revealed by CTCA.

The exclusion criteria are:

1. Noncoronary etiology of angina, for example, anemia, aortic stenosis, and hypertrophic obstructive cardiomyopathy.
2. Obstructive coronary disease evident in an artery (diameter >2.5 mm), that is, >50%-70% circumferential plaque extending for ≥2 coronary segments or a stenosis >70% as revealed by CTCA.
3. Lack of informed consent.

Exclusion for the randomized study only:

1. Flow-limiting coronary disease defined by FFR ≤ 0.80 in an artery with a diameter of more than 2.5 mm.

CTCA will be performed during usual care and acquired according to a standard protocol. Where preliminary noncontrast scans are acquired, CT coronary calcium score will be estimated according to local practice. Oral and/or intravenous β-blocker therapy (if required for heart rate control) and sublingual glyceryl trinitrate (GTN) will be given immediately prior to CTCA in line with local standards of care. The CTCA should be of sufficient diagnostic quality to substantiate a conclusive radiology report whereby disease severity in an epicardial coronary artery with a diameter> 2.5 mm is classified by the reporting clinician as absent or present and, if present, whether the disease is obstructive, that is, >70% severity; potentially obstructive, >50%-70% circumferential with plaque extending for ≥2 coronary segments; intermediate, >50%-70% plaque but not circumferential plaque extending for severity; or nonobstructive CAD (≤50%). This classification aligns with contemporary trials.8., 10., 11., 12., 13., 18. Overall disease severity will be categorized using the CAD-RADS reporting system for stable chest pain.^25^

Participants without obstructive CAD on CTCA will continue in the study. They will be invited to attend on a different date for elective coronary angiography with adjunctive tests of coronary function. These procedures will be performed in a single reference center (Golden Jubilee National Hospital). During the angiogram, participants either with obstructive CAD or who are eligible but for other reasons, for example, logistical, are not randomized will continue in a follow-up registry (Figure 2). The Research Ethics Committee and Research and Development Management Office have approved the protocol.

### Randomization and implementation

The treatment plan will be serially recorded by the attending cardiologist before and after coronary angiography but before randomization in the catheter laboratory. The noninvasive CTCA findings will be reevaluated using invasive coronary angiography and guidewire-based FFR in any major coronary artery with CAD >50% of the reference vessel diameter. Participants who have flow-limiting CAD will be considered for revascularization by percutaneous coronary intervention or coronary artery bypass graft surgery, as appropriate.

The participants with no obstructive CAD (FFR >0.80) will undergo testing of coronary function using an IDP. The participants will be randomly assigned 1:1 to the intervention (stratified medicine according to IDP results) or blinded control (IDP performed but results not disclosed [sham procedure]; standard-care medical therapy according to physician preference). The results will be disclosed to the attending cardiologist in the intervention group and not disclosed in the control group. In the intervention group, the cardiologist can reappraise the diagnosis based on coronary angiography and can change the diagnosis with linked therapy decisions. In the control group, management will be guided by coronary angiography and the other available medical information but not the IDP results. Guideline-directed medical management will be implemented.^17^ The attending clinicians will be provided with a standardized management document (Table I) that is specific to the final diagnosis (endotype). The same approach will be implemented for participants in both groups. The management in the intervention group reflects stratified medicine with treatment linked to the endotype (intervention group, IDP disclosed). Management in the control group reflects angiography-guided medical management (IDP not disclosed).

**Table I:** Endotypes: definitions and guidance therapy for attending cardiologists

Diagnostic group/endotype		Outcome definitions	Linked therapy
Microvascular angina	Increased microvascular resistance	IMR >25 IMR is a quantitative method for specifically assessing microvascular function independent resting hemodynamics.	**Baseline therapy**: consider aspirin, statin, and ACE-i therapy in all patients. Sublingual GTN as required. **Antianginal therapy** (except microvascular spasm): ***1st line*** β-Blocker (eg, carvedilol 6.25 mg twice daily, to be uptitrated) ***2nd line*** Non-DHP CCBs substituted (eg, verapamil 120 mg slow release) where β-blockers are not tolerated or ineffective. ***3rd line*** (add in therapy) DHP CCB (eg, amlodipine) for those on β-blockers Nicorandil (5 mg twice daily, to be uptitrated) Ranolazine (375 mg twice daily, to be uptitrated) **Antianginal therapy** (microvascular spasm only): treat like vasospastic angina (see below) Refer for cardiac rehabilitation
	Reduced coronary vasorelaxation	CFR <2 This reflects the inability to increase coronary flow above 2× the resting flow.	
	Reduced microvasodilator capacity	RRR <2 This reflects the vasodilator capacity of the microcirculation to change from baseline to hyperemia	
	Microvascular spasm	Angina with typical ischemic ECG changes and epicardial coronary constriction <90% reduction in epicardial coronary artery diameter during ACh infusion. This represents increased microvascular constriction.	
Vasospastic angina	Epicardial spasm	Epicardial coronary artery spasm (>90% reduction in coronary diameter) with symptoms and ST-segment changes following IC ACh in comparison with baseline resting condition following IC GTN administration in any epicardial coronary artery segment.	**Baseline therapy**: Aspirin and statin should be considered. PRN sublingual GTN **Antianginal therapy**: ***1st line*** Non-DHP CCB (eg, verapamil 120 mg slow release, to be uptitrated) ***2nd line*** (add in therapy) Add nitrate, eg, isosorbide mononitrate 10 mg BD ***3rd line*** Change nitrate to nicorandil (5 mg twice daily, to be uptitrated) Refer for cardiac rehabilitation
Obstructive epicardial stenosis		FFR ≤ 0.80	**Baseline therapy**: Aspirin and statin should be considered. Sublingual GTN as required. Consideration of revascularization, antianginal therapy as per ESC guidelines. Refer for cardiac rehabilitation
Noncardiac chest pain		FFR >0.80 CFR ≥2 RRR ≥2 IMR ≤25 No functional angina/spasm during ACh infusion	Cessation of antianginal therapy. Stop antiplatelet and statin unless other indication present. Consider noncardiac investigation or referral where appropriate.

*RRR*, resistance reserve ratio; *ACh*, acetylcholine; *ACE-I*, angiotensin-converting enzyme inhibitor; *DHP*, dihydropyridine; *CCB*, calcium channel blocker; *IC*, intracoronary; *ESC*, European Society of Cardiology.

Catheter laboratory staff will use a Web-based randomization tool to immediately randomize the patient after the index coronary angiography revealed no obstructive CAD (Robertson Centre for Biostatistics, University of Glasgow). The randomization sequence involves block lengths randomized in blocks of length 4, that is, every 20 allocations consists of 4 blocks, 2 of length 4 and 2 of length 6, in a random order. Patients will be considered as being randomized as soon as the allocation is assigned on the Web-based portal.

### Blinding and adherence

Patients in the control arm will undergo the IDP in the same way as the participants in the intervention group except that the results will not be disclosed to the treating cardiologist in the control group. Blinding will be implemented by obscuring the hemodynamic monitor from the clinicians, nurses, and participants such that it will be impossible for them to observe the IDP results. Complete blinding will be ensured through the assistance of a second cardiologist (N. S.) who supervised the coronary function and vasoreactivity testing protocol. The attending cardiologist will be invited to leave the catheter laboratory for the duration of the diagnostic procedure. For this reason, it is not possible to blind the cardiologist to the randomized group allocation. The cardiologist and the participant will remain blinded to the diagnostic findings in the control group. Pharmacological tests will be performed in an identical fashion in both groups. Adherence to monitoring and blinding will be prospectively recorded by the research staff. The outcome assessors and statisticians will be blinded to treatment group allocation.

### Coronary function testing (IDP)

The stratified medicine protocol^9^ is supported by contemporary practice guidelines.^17^ On practical grounds, the IDP will be performed in a single major coronary artery to curtail the duration of the procedure. The left anterior descending coronary artery will usually be the target vessel because it supplies the greatest amount of ventricular mass. The decision will be at the discretion of the interventional cardiologist. If the IDP test results are normal and clinical suspicion remains high, then additional arteries may be assessed, in line with clinical judgement.

The IDP involves a coronary thermodilution technique. A pressure- and temperature-sensitive diagnostic coronary guidewire will be advanced into a major coronary artery (typically into the left anterior descending coronary artery) for assessment of CFR (abnormal <2.0), IMR (abnormal >25), and FFR (abnormal ≤0.80) during intravenous infusion of adenosine (140 μg/kg/min).

Incremental concentrations of acetylcholine (10^−6^, 10^−5^, and 10^−4^ mol/L) will then be sequentially infused during 2-minute periods, followed by vasospasm provocation testing (acetylcholine bolus, 100 μg for left coronary artery or 50 μg right) and finally 300 μg of glyceryl trinitrate. An angiogram will be acquired at the end of each infusion period.

### Definitions of endotypes

The IDP will be used by the attending cardiologist to assess for coronary endotypes according to diagnostic criteria defined in practice guidelines.17., 26., 27. Considering the randomized trial, in the intervention arm, the IDP will be used to stratify patients into subgroups (endotypes: microvascular angina, vasospastic angina, both, none, or flow-limiting CAD [an exclusion criterion]). The diagnosis of a clinical endotype will be linked to guideline-based management.^17^ A diagnosis of vasospastic angina requires that 3 conditions be satisfied during acetylcholine testing: (1) clinically significant epicardial vasoconstriction (≥90%), (2) reproduction of the usual chest pain and, (3) ischemic electrocardiographic (ECG) changes.^27^ Microvascular angina is defined according to standardized Coronary Vasomotion Disorders International Study Group diagnostic criteria^26^: symptoms of myocardial ischemia, unobstructed coronary arteries, and evidence of coronary microvascular dysfunction (any of abnormal IMR, CFR, or microvascular spasm to acetylcholine). A diagnosis of coronary microvascular spasm requires provocation and reproduction of anginal symptoms and ischemic ECG shifts but no epicardial spasm during acetylcholine testing.^26^ A diagnosis of noncardiac chest pain requires no obstructive epicardial CAD (FFR >0.80) and an absence of evidence of any functional coronary disorder (CFR >2.0, IMR <25, and negative acetylcholine testing).

### Stratified medicine in the intervention group

After randomization and completion of the diagnostic intervention, research staff will invite the cardiologist to consider the new findings and reevaluate the diagnosis and treatment plan initially made based on coronary angiography. The attending cardiologist will be provided with written management guidance enabling a personalized medicine approach linked to the endotype and informed by practice guidelines.^17^ Standardized guidance letters will be provided to the general practitioner and attending cardiologist with advice on tailoring and optimizing treatment (including nonpharmacological and lifestyle measures). Standard care for participants in the control arm consists of guideline-directed medical therapy. The attending cardiologist has discretion over the final treatment decisions in both groups.

### Questionnaires and follow-up

The SAQ is a self-administered, disease-specific measure of angina severity that is valid, reproducible, and sensitive to change.^24^ The SAQ quantifies patients' physical limitations caused by angina, the frequency of and recent changes in their symptoms, their satisfaction with treatment, and the degree to which they perceive their disease to affect their quality of life. Each scale is transformed to a score of 0 to 100, where higher scores indicate better function (eg, less physical limitation, less angina, and better quality of life). The summary score (SAQSS) averages the domains of angina limitation, frequency, and quality of life to provide an overall metric of angina severity.^24^ In the CorMicA trial, the SAQ disclosed differences between the randomized groups.^9^

Health status will be serially assessed using validated, self-administered questionnaires for quality of life using the EuroQOL (EQ-5D-5L). This is a widely used standardized instrument for measuring generic health status whereby higher scores represent better health-related quality of life (from −0.59 to 1.00 scale).28., 29. We will also record the Brief Illness Perception Questionnaire,^30^ screening for depression and anxiety (Patient Health Questionnaire-4),^31^ and the Treatment Satisfaction Questionnaire for Medication.^32^ At 6 and 12 months, patients' anginal symptoms will be reassessed using the same questionnaires.

#### Sample size calculation and statistical analysis for the diagnostic study

The primary analysis will be the between-group comparison of the reclassification rate using logistic regression, adjusted for baseline characteristics associated with the likelihood of reclassification of the initial diagnosis. Prespecified baseline characteristics are anticipated to include sex and smoking status. If this is not possible because of small numbers, logistic regression with fewer adjustment variables or Fisher exact test will be used as appropriate. A sample size of 115 per group will have 80% power to detect a between-group difference of 15%, or 90% power to detect a difference of 20%, in the proportion of patients whose diagnosis is reclassified. To allow for any missing data, 250 patients will be randomized (Table II). If the coronary function test results are disclosed in the usual-care group (operator preference; protocol deviation), the plan before disclosure will be recorded.

**Table II:** Sample size calculations for the diagnostic study

Sample size calculation: change in diagnosis (% of patients)		Power, %	Group size, n
Disclosed group	Usual-care group		
10	0.1⁎	90	94†
20	0.1⁎	90	45†
20	5	90	114
25	5	90	75
25	10	80	113
30	10	90	92

⁎ Approximately 0%-1% reclassification.

† Calculation based on Fisher exact test.

#### Sample size calculation for the randomized controlled trial

If SAQ scores at 6 months can be obtained from 180 patients (72%), the trial will have 80% power to detect a mean between-group difference in within-subject change in SAQ scores of 0.42 SD unit. This is a small difference, but we anticipate that not all patients will have their therapy changed following disclosure of the IDP result. Using the coronary function data for the control (nondisclosure) group, we will carry out focused analyses of the subgroup of patients whose therapy might have been altered based on abnormal results. For example, if therapy would be altered in 50% of patients, the study will have 80% power to detect a difference in SAQ score of 0.60 SD unit for these patients; if therapy is altered in 30% of patients, there will be 80% power to detect a between-group difference of 0.74 SD unit. We anticipate loss to follow-up in ≤15% of the participants. The sample size is suggested to be sufficiently large to limit imprecision and be clinically meaningful.

### Statistical analysis of the randomized controlled trial

Continuous outcomes at 6 months will be compared between study groups using linear regression adjusting for stratification variables used in the randomization (site, sex, and CAD on CT scan) and baseline level of the outcome where applicable. Where residuals are clearly not normally distributed, standard transformations will be applied to the outcome prior to analysis to achieve approximate normal distribution of the residuals. In addition, changes from baseline at all follow-up measurements will be analyzed over time using linear mixed-effects regression models. Time-to-event outcomes will be compared between treatment groups using Cox proportional hazards regression adjusting for stratification variables used in the randomization provided the proportional hazards assumption is met. Otherwise, log-rank tests will be used.

### Follow-up procedures

Follow-up assessments for adverse events will be performed by the clinical research staff by telephone or in person (eg, outpatient clinic review), as appropriate. Medical records will also be checked. Follow-up contact will occur at 6 monthly intervals until the last patient has achieved a minimum of 6 months of follow-up. Follow-up in the longer term (ie, ≥3 years) will be supported by electronic record linkage with central government health records. The active phase of the project will be completed within 30 months. Follow-up procedures will be the same for patients in both groups. The adherence to blinding will be prospectively recorded and monitored. The participants’ knowledge of their treatment group assignment will be checked at 12 months.

Written management guidance for each endotype, informed by practice guidelines, will be provided to the cardiologist, GP, and nurse practitioners with advice to start, stop, and optimize treatment (including nonpharmacological/lifestyle measures) in line with the final diagnosis.

#### Trial management and governance

The study will be conducted according to observational (STROBE),^33^ GCP,^34^ and CONSORT^35^ guidelines. The study will be coordinated by the Study Management Group that includes those individuals responsible for the day-to-day management of the study including the Chief Investigator, Co-Investigators, Research Nurse, and others as considered appropriate. The role of this group will be to facilitate the progress of the study, ensure that the protocol is adhered to, and take appropriate action to safeguard participants and the quality of the study itself.

Clinical events identified as potentially relevant to the designated secondary health outcomes will be assessed by a Clinical Event Committee. The Clinical Event Committee will be independent of both the investigators and the funder/sponsor and will be blinded regarding any information relating to the randomization group. Study monitoring will be conducted by monitors on behalf of the sponsor (NHS Golden Jubilee National Hospital). During monitoring assessments, informed consent forms and source clinical data will be reviewed as appropriate.

### Source of funding

This research was supported by the British Heart Foundation (BHF) and the Chief Scientist Office of the Scottish Government.

## Discussion

Our study will provide new information on the prevalence and clinical significance of microvascular angina and vasospastic angina in a comparatively unselected population of patients with angina and no obstructive CAD classified by clinically indicated CTCA. The trial includes multiple centers, all of which participated in the SCOT-HEART trial.8., 11. The study design includes measures to mitigate against bias. Overall, the study is intended to have transferable relevance to clinical practice and guidelines.

A clinical strategy of anatomical imaging of CAD exploits the high negative predictive value of CTCA and the benefits of identifying coronary atherosclerosis to stratify affected patients for preventive medical therapy. Subjects with obstructive CAD can also be referred for invasive management. However, this strategy does not take account of myocardial ischemia which is the downstream consequence of reduced coronary blood flow due to either an anatomical or functional problem, or both. Patients with anginal symptoms and no obstructive CAD classified by CTCA may have myocardial ischemia due to either a false-negative test result, that is, flow-limiting CAD (FFR ≤0.80), or ischemia due to microvascular angina, vasospastic angina, or both. Microvascular angina typically causes effort- and stress-induced angina. Vasospastic angina typically occurs spontaneously or in response to triggers such as exertion, emotional stress, or cold weather. Affected patients may experience spontaneous and recurrent episodes of angina. Patients with vasospastic angina may have a true-negative stress test result for myocardial ischemia.

The emergence of diagnostic guidewire-based tests of coronary vascular function opens the door to assessing endotypes in clinical practice.9., 17. The strategy is now supported by a Class IIA recommendation from the European Society of Cardiology.^17^ Specifically, IMR is informative for structural microvascular disease, and CFR is informative for functional vasomotion disorders. IMR and CFR have prognostic significance, including in INOCA when flow-limiting CAD is ruled out.^36^ Furthermore, the diagnostic guidewire test complements adjunctive provocation testing with acetylcholine — together, these physiological parameters may predict propensity to adverse cardiac events.^37^ Coronary reactivity testing with acetylcholine is used to assess for vasospasm. This approach is also supported by a Class IIA guideline recommendation.^17^

Most patients with recent-onset angina do not have obstructive CAD. In SCOT-HEART,^8^ just 25% of the participants had obstructive CAD (>70% stenosis in ≥1 major branches or 50% in the left main stem). In PROMISE,^10^ only 517 (10.7%) of the 4,996 participants in the CTCA group had obstructive CAD. Both of these trials had a pragmatic design, and invasive management was not systematically performed, leaving uncertain the etiology of the angina in the majority of the participants in these trials, notably the patients without obstructive CAD. The CE-MARC 2 trial compared 3 diagnostic strategies ([1] CMR, [2] myocardial perfusion scintigraphy group, and [3] NICE guidelines involving CTCA) in 1,202 patients with angina referred to the Chest Pain Clinic with a pretest likelihood of CAD of 10% to 90%.^38^ Invasive coronary angiography was performed within 12 months of randomization in 265 (22%) patients. The primary outcome of unnecessary angiography (defined as an FFR >0.8 or quantitative coronary analysis showing no stenosis ≥70% in 1 view or ≥50% in 2 orthogonal views in all coronary vessels ≥2.5 mm diameter) occurred in 139 subjects (12%): 7.5% in the CMR group, 7.1% in the myocardial perfusion scintigraphy group, and 28.8% of participants in the NICE guidelines group.^38^ The CE-MARC 2 physiology substudy highlighted that microvascular angina may be relevant, but invasive tests were not systematically performed.^39^

The ISCHEMIA trial investigators^40^ observed that some of the participants enrolled with moderate-severe myocardial ischemia on stress testing (% LV mass) do not have obstructive CAD. The Changes in Ischemia and Angina Over 1 Year Among ISCHEMIA Trial Screen Failures With no Obstructive CAD on Coronary CT Angiography (CIAO) ISCHEMIA study will investigate ischemia in these patients using stress echocardiography (ClinicalTrials.gov Identifier: NCT02347215).

In FAME-2,41., 42. of 1,220 patients with stable CAD, 332 (27%) had non–flow-limiting (FFR >0.80) CAD (registry group). The distribution of the Canadian Cardiovascular Society angina classes was similar between the randomized and medically managed registry populations (*P* = .64), as was the prevalence of silent ischemia (16%; *P* = .96). The MACE rate in the registry group was 9% at 2 years, highlighting the prognostic implications of non–flow-limiting CAD.^43^

The CorCTCA trial is designed to extend the evidence from these trials and fill key knowledge gaps. CorCTCA should clarify the prevalence and clinical significance of vasomotion disorders in INOCA. Considering the potential clinical implications, anatomical imaging with CTCA as a first-line test may lead to missed diagnoses of microvascular angina and vasospastic angina, and suboptimal outcomes for patients with these conditions. In SCOT-HEART, CTCA was associated with an increase in anginal symptoms and reduction in quality of life.^19^ These outcomes were most pronounced in the patients with nonobstructive CAD. There could be several reasons to explain this finding. Firstly, patient satisfaction may be greater with a definitive diagnosis and treatment plan, that is, (1) normal coronaries = stop treatment and (2) obstructive CAD = percutaneous coronary intervention or coronary artery bypass graft surgery, whereas a result of no obstructive CAD and no change in treatment may reduce patient satisfaction. Secondly, a false-negative CTCA result may have resulted in some patients with flow-limiting CAD not being referred for invasive management. An alternative strategy involving functional tests, for example, FFR-CT or ischemia testing, would reduce this problem.^44^ Finally, some patients may have had microvascular disease, and discontinuation of angina treatment by protocol may have led to a deterioration symptoms. In light of practice guideline recommendations,15., 16., 17. the community adoption of anatomical imaging with CTCA as a first-line test for the assessment of stable chest pain14., 15., 16. increases the relevance of our research, not least because guideline-directed therapy is recommended for microvascular angina and vasospastic angina.^17^

The CorCTCA protocol presents some ethical considerations. First, a coronary angiogram exposes the participants to ionizing radiation. Second, the angiogram is invasive. Third, following a “negative” CTCA scan result, the standard of care is typically for discharge from cardiology review. These points should be balanced against the possibility of false-negative CTCA results and the potential benefits of this research. Quality of life,9., 19. anginal symptoms,9., 19. and prognosis9., 36., 43. remain impaired when management is guided by anatomical CTCA. Clinical audit and prospective studies^9^ indicate persisting service user dependency. In the National Health Service, about one quarter of patients referred for elective coronary angiography have previously had an angiogram,^9^ and repeated attendance in primary and secondary care by patients with INOCA is common, yet potentially avoidable.^17^ These patients have an unmet need, and most are female,^8^ raising questions around the optimal diagnostic management of IHD in women.^45^ Because CTCA is an anatomical test, the report may underestimate the true severity of CAD, and some patients with flow-limiting CAD may not be referred for revascularization.

We do not propose that invasive testing of coronary function should be indicated in all patients with angina and a “negative” CTCA scan result, rather that it may be a helpful option for patients when persistent symptoms are unexplained by noninvasive testing. Our study will provide information on whether noninvasive functional testing should be a primary test option in line with recent guidelines.^17^
